# Enhanced anti-tumor activity and cytotoxic effect on cancer stem cell population of metformin-butyrate compared with metformin HCl in breast cancer

**DOI:** 10.18632/oncotarget.9522

**Published:** 2016-05-20

**Authors:** Kyung-Min Lee, Minju Lee, Jiwoo Lee, Sung Wuk Kim, Hyeong-Gon Moon, Dong-Young Noh, Wonshik Han

**Affiliations:** ^1^ Biomedical Research Institute, Seoul National University Hospital, Seoul 110-744, Republic of Korea; ^2^ Cancer Research Institute, Seoul National University College of Medicine, Seoul 110-744, Republic of Korea; ^3^ Department of Surgery, Seoul National University College of Medicine, Seoul 110-744, Republic of Korea; ^4^ Hanall Biopharma Co., Ltd., Seoul 138-922, Republic of Korea

**Keywords:** metformin analog, breast cancer, stem cell population, G2/M arrest, anti-cancer drug

## Abstract

Metformin, which is a drug commonly used to treat type 2 diabetes, has shown anti-tumor effects in numerous experimental, epidemiologic, observational, and clinical studies. Here, we report a new metformin derivative, metformin-butyrate (MFB). Compared to metformin-HCl, it more potently activates AMPK, inhibits mTOR, and impairs cell cycle progression at S and G2/M phases. Moreover, MFB inhibits the mammosphere formation of breast cancer cells and shows cytotoxic effects against CD44^+^CD24^−/low^ populations *in vitro* and *in vivo*, indicating that it might have preferential effects on the cancer stem cell population. MFB showed synergistic cytotoxicity with docetaxel and cisplatin, and MFB pretreatment of breast cancer cells prior to their injection into the mammary fat pads of mice significantly decreased the obtained xenograft tumor volumes, compared with untreated or metformin-pretreated cells. Overall, MFB showed greater anti-neoplastic activity and greater efficacies in targeting the G2/M phase and breast cancer stem cell population, compared to metformin-HCl. This suggests that MFB may be a promising therapeutic agent against aggressive and resistant breast cancers.

## INTRODUCTION

Metformin (1, 1-dimethylbiguanide hydrochloride), which is the most widely used anti-diabetic drug for type 2 diabetes, has recently been shown to possess strong anti-cancer effects [1]. Recent epidemiologic studies have indicated that the use of metformin in type II diabetic patients significantly suppresses the development of cancers and lowered cancer-related mortality [1–3]. Furthermore, a retrospective study found that the addition of metformin to neoadjuvant chemotherapy yielded a significantly higher rate of pathologic complete response (pCR) in diabetic patients with breast cancer [4]. Preclinical studies in animal model systems have shown that metformin decreases the tumor growth of breast [5, 6], colon [7], and pancreatic [8] cancers. Therefore, metformin appears to be an effective means of dealing with cancer. Mechanistically, metformin has been shown to affect AMPK and mTOR signaling activity [9–13] and modulate cell cycle-related molecules to cause G1 arrest and apoptosis [14–16].

The abilities of anti-cancer drugs to cause cell growth arrest and/or apoptosis are often conferred via modulation of cyclins, cyclin-dependent kinases (CDKs) and/or cyclin-dependent kinase inhibitors (CKIs), leading to G1 or G2/M arrest [17, 18]. G1-phase cyclin D and CDK4/6 can phosphorylate the retinoblastoma protein (pRB) to release E2F, triggering transcriptional activation of the cyclin E promoter as the cell passes through the restriction point prior to S phase [18]. The active cyclin E-CDK2 complex then further phosphorylates pRB to enhance the E2F-dependent transcriptional activation of cyclin A promoter regions, thereby enabling efficient S phase progression [18, 19].

The cancer stem cell hypothesis postulates that tumors are maintained by self-renewing cancer stem cells (CSCs; also called tumor-initiating cells) that are also capable of differentiating into non-self-renewing cell populations for the bulk tumor mass [20, 21]. CSCs have the stem cell characteristics of self-renewal or tumor-initiation, and may play critical roles in tumorigenic growth, metastasis, and therapeutic resistance [22–25]. To improve the efficacy of cancer therapy, it could be useful to develop novel therapeutic reagents that can eliminate or target CSCs [23, 25]. Although recent studies have implicated metformin as a therapeutic agent capable of targeting CSCs [5, 26–30], the mechanism(s) underlying the inhibitory effects of metformin on CSCs are not completely understood. Moreover, a major potential limitation in using metformin as an anti-cancer reagent is the high effective concentration of metformin during determination of metformin concentration that can be safely treated during *in vitro* analyses and in the (pre)clinical purposes [13, 31]. To address these issues, we hypothesized that it could be possible to develop a structural analog of metformin that could have an even more potent anti-cancer activity and target specificity than metformin.

In our efforts to develop novel metformin derivatives with increased potency for AMPK activation and mTOR inhibition, we found that the anti-cancer effects of metformin-butyrate (MFB) appeared to outperform those of metformin at lower doses. Compared to metformin, MFB had much lower (2 ~ 30-fold) IC_50_ values for triggering G1 and G2/M arrest, impairing S phase entry and/or progression, and inducing apoptosis *in vitro* and *in vivo*. Further, MFB showed more potent targeting of the CD44^+/high^/CD24^−/low^ CSC-like population compared to metformin. These results suggest that MFB could be a promising anti-cancer reagent that is capable of targeting S phase events with greater specificity compared to metformin.

## RESULTS

### A novel metformin derivative (MFB) shows more potent anti-proliferative effects on breast cancer cells than metformin

**Figure 1:**
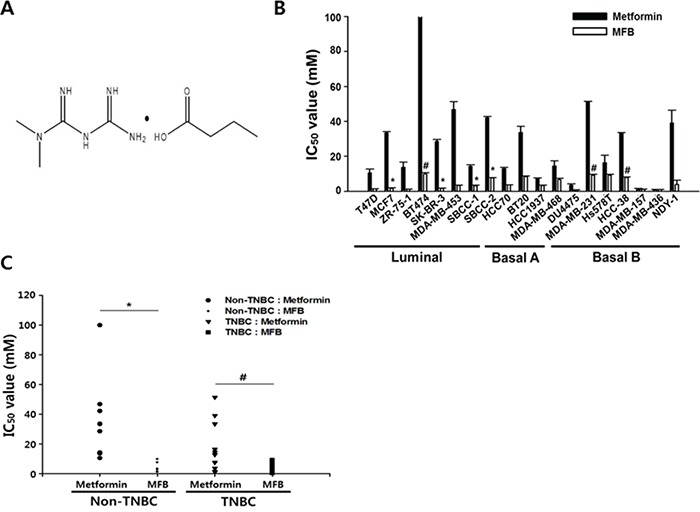
Compared to metformin, metformin-butyrate (MFB) shows more potent anti-proliferative effects on breast cancer cells **A.** The chemical structure of MFB. **B** and **C.** Cytotoxicities of metformin and MFB (0.01-100 mM) against various breast cancer cell lines, as determined using a CellTiter-Glo assay. The average IC_50_ values (the half-maximal inhibitory concentrations) were graphed for metformin or MFB in different breast cancer subtypes (B) and TNBC vs. non-TNBC cells (C) for 48 h. Symbols: bars, SD; *, *p* < 0.05; ^#^, *p* < 0.01 (versus metformin). The data shown are representative of three independent experiments.

To identify the metformin derivatives with the highest cytotoxicity against cancer cells, we screened a series of metformin analogs for their ability to decrease viability in breast cancer cell lines (data not shown). Nineteen breast cancer cell lines, including the luminal and basal A and B subtypes (also termed triple-negative breast cancer, TNBC), were treated with 0.01 to 100 mM of each metformin analog for 48 h, and cytotoxicity was compared to that conferred by metformin (positive control). Among the tested metformin derivatives, MFB was selected as the most effective anti-cancer compound (Figure 1A). In the metformin-resistant (defined as an IC_50_ value of > 50 mM) BT474 luminal A breast cancer cell line, MFB exerted approximately 10-fold more potent cytotoxic effects than metformin (IC_50_, 9.1 mM versus > 100 mM, respectively; Figure 1B). MFB also exerted more potent cytotoxic effects against MDA-MB-453 Her2-positive breast cancer cells (IC_50_, 3.4 mM) and MDA-MB-231 TNBC (IC_50_, 9.2 mM) compared to metformin (IC_50_, 51.3 mM and 51.4 mM, respectively) (Table 1 and Figure 1C).

**Table 1:** The IC_50_ values for metformin or MFB in various breast cancer cell lines

Cell Lines	IC _50_ value		Gene	ER	PR	HER2	Soruce	Tumor Type
	Metformin	MFB	cluster					
T47D	12.6	1.5	Lu	+	+	−	PE	IDC
MCF7	33.4	1.5	Lu	+	+	−	PE	IDC
ZR-75-1	10.7	1.2	Lu	+	−	−	AF	IDC
BT474	100	9.1	Lu	+	+	+	P. BR	IDC
SK-BR-3	28.4	1.5	Lu	−	−	+	PE	AC
MDA-MB-453	51.3	3.4	Lu	−	−	+	PE	AC
SBCC-1	13.3	2.6	Lu	−	−	+	P. BR	n.d.
SBCC-2	41.4	7.7	Lu	+	−	−	P. BR	n.d.
HCC70	12	3.6	Ba A	−	−	−	P. BR	Duc. Ca
BT20	33.6	8.5	Ba A	−	−	−	P. BR	IDC
HCC1937	7.4	3.4	Ba A	−	−	−	P. BR	Duc. Ca
MDA-MB-468	14.4	6.7	Ba A	−	−	−	PE	AC
DU4475	3.6	0.5	Ba A	−	−	−	SK	IDC
MDA-MB-231	51.4	9.2	Ba B	−	−	−	PE	AC
Hs578T	16.3	4.2	Ba B	−	−	−	P. BR	IDC
HCC-38	31.6	7.8	Ba B	−	−	−	P. BR	Duc. Ca
MDA-MB-157	1.5	1.2	Ba B	−	−	−	PE	MC
MDA-MB-436	1.1	1.1	Ba B	−	−	−	PE	IDC
NDY-1	31.6	1.5	Ba B	−	−	−	P. BR	SAR

Lu, luminal; PE, pleural effusion; IDC, invasive ductal carcinoma; P.ER, primary breast; AC, adenocarcinoma; BaA, Basal A; BaB, Basal B; SK, skin; Duc. Ca; ductal carcinoma; MC, metaplastic carcinoma; SAR, sarcoma; n.d. not defined

No significant difference in cytotoxicity was observed in metformin-sensitive cell lines (e.g., MDA-MB-157 and MDA-MB-436 cells) treated with metformin versus MFB, but MFB generally exhibited 2- to 30-fold greater cytotoxicity against most of the tested breast cancer cell lines (Figure 1B and 1C). Our results suggest that MFB can inhibit cancer cell proliferation, and that these effects are more potent than those of metformin. Notably, metformin was preferentially cytotoxic to TNBC cells, showing mean IC_50_ values of 31.2 mM for non-TNBC (luminal subtype) cells and 17.2 mM for TNBC cells. In contrast, the mean IC_50_ values of MFB did not differ between these groups (4.3 and 4.1 mM, respectively) (Figure 1C). This suggests that, compared to metformin, MFB exerts stronger cytotoxic activities against breast cancer cells, regardless of the subtype.

### MFB treatment of breast cancer cells causes aberrant S phase progression and/or apoptosis

**Figure 2:**
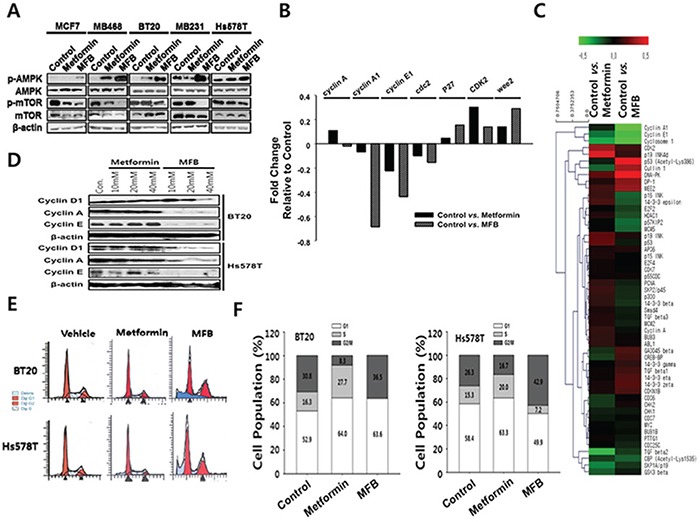
MFB treatment of various breast cancer cells causes aberrant S phase progression and/or apoptosis **A.** Subconfluent cultures of breast cancer cell lines were treated with 10 mM of metformin or MFB for 18 h, and the cells were collected and subjected to Western blot analyses with specific antibodies against AMPK, p-AMPK, mTOR, p-mTOR and ß-actin. **B.** A heat map of cell cycle-related proteins on metformin or MFB treated MDA-MB-231 cell line. The heat map represents the most significant protein (cell cycle-related) changes in all the comparison groups (vehicle *vs*. metformin, vehicle *vs*. MFB). Red, increased protein expression; green, low relative to the other samples. **C.** Representative results of antibody array analysis of cell-cycle-relative molecules in metformin- or MFB-treated MDA-MB-231 cell lines. **D-F.** Subconfluent BT20 and Hs578T cells were treated with 10 mM metformin or MFB for 24 h. Whole-cell lysates were analyzed for cyclin A, cyclin E and D1 by Western blotting (D), and cell cycle analysis was performed via flow cytometry following propidium iodide (PI) staining (E and F).

Since metformin is known to affect AMPK/mTOR signaling activity [32], we compared the effects of MFB and metformin in this regard. Various breast cancer cell lines (luminal type, MCF7 and BT474; TNBC type, BT20, MDA-MB-231, Hs578T and NDY-1) were treated with metformin or MFB (10 mM) for 12 h, and cell lysates were analyzed for their levels of p-AMPK (T172) and p-mTOR (S2448). Our results revealed that MFB activated AMPK and inhibited mTOR with significantly greater potencies than metformin in all of the tested breast cancer cell lines (Figure 2A). This suggests that MFB could potentiate the pharmacological activities of metformin.

To test whether these two drugs have different effects on cell cycle progression, we used an antibody array to analyze cell cycle-related molecules (Figure 2B) in extracts from metformin- or MFB-treated MDA-MB-231 cells. Our results revealed that MFB treatment decreased cyclin A, E and cdc2 (which are related to S phase entry and progression) and increased Wee2 (a negative regulator of CDK2/cyclin B complex during the G2 to M phase transition) significantly more than did metformin treatment (Figure 2B and 2C). Immunoblotting showed that while treatment with metformin at concentrations up to 40 mM did not change the levels of cyclins D, E or A, treatment with 20 mM MFB diminished the levels of these cyclins; indeed, treatment with 10 mM MFB rendered cyclin A almost completely undetectable (Figure 2D). This observation suggests that MFB impairs S phase entry and/or progression more effectively than metformin. Next, we used flow cytometry to analyze the proportions of cells in each of the cell cycle phases following treatment with or without metformin or MFB. Compared to the vehicle controls, BT20 and Hs578T cells treated with MFB (10 mM) for 24 h showed almost no S phase cells and a significant proportion of cells were arrested in G2/M phase, whereas those treated with metformin were mostly arrested in G1 phase (Figure 2E and 2F). These findings indicate that MFB appears to target S phase entry and/or progression through G2/M phase more specifically than does metformin.

**Figure 3:**
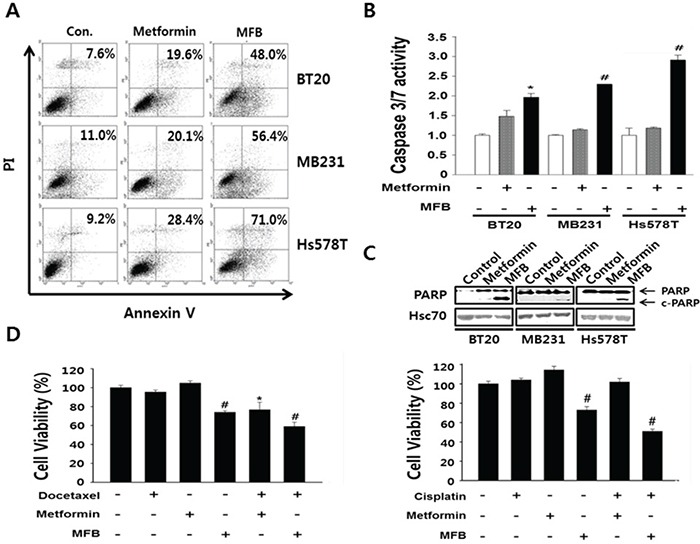
MFB induces the apoptosis in breast cancer cells **A.** Subconfluent BT20, MDA-MB-231 and Hs578T cells were treated with 10 mM metformin or MFB for 48 h and analyzed for cellular apoptosis by Annexin V/PI staining followed by flow cytometry. Apoptosis were confirmed Caspase-3/7 activities using the Caspase-Glo 3/7 assay **B.** and c-PARP expression **C. D.** BT20 (left) and MDA-MB-231 (right) cells were treated with 10 mM metformin or MFB in the presence or absence of docetaxel (50 nM) or cisplatin (50 μM) for 48 h, and cell viability was analyzed using a CellTiter-Glo Luminescent Cell Viability Assay Kit. Data are expressed as the means ± SD of triplicate experiments. Symbols: *, *p* < 0.05; ^#^, *p* < 0.01 compared to controls. The data shown are representative of three different experiments.

To examine whether MFB could induce apoptosis more effectively than metformin, we performed flow cytometric analysis of Annexin V-stained TNBC cell lines treated with or without metformin or MFB. Our results revealed that, compared to metformin, MFB triggered a greater degree of apoptosis in the tested cell lines (Figure 3A). Following treatment with 10 mM MFB, the apoptotic populations increased as follows: BT20 cells, from 7.6% (before treatment) to 48% (after treatment); MDA-MB-231 cells, 11% to 56.4%; and Hs578T cells, 9.2% to 71% (Figure 3A). As an additional indication of apoptosis, we measured caspase-3/7 activity in MDA-MB-231 cells. Interestingly, MFB-treated cells showed up to 3-fold increases in caspase-3/7 activity, whereas the metformin-treated cells did not (Figure 3B). These results were further confirmed by an increase in the cleaved (active) form of caspase-3 (data not shown). MFB treatment also increased poly (ADP-ribose) polymerase 1 (PARP1) cleavage to a greater degree than metformin treatment (Figure 3C). These results suggest that MFB treatment more induces caspase- and/or PARP-mediated apoptosis in breast cancer cells than metformin. We next tested the effect of co-treating cells with MFB plus cisplatin or docetaxel, which are the most widely used chemotherapeutic agents for breast cancer. Co-treatment of BT20 (docetaxel-resistant) or MDA-MB-231 (cisplatin-resistant) cells with 5 mM MFB plus 50 nM docetaxel or cisplatin yielded significantly greater cytotoxic effects compared to the co-treatment of cells with metformin plus either chemotherapeutic agent (Figure 3D). This suggests that drug resistance could be overcome by co-treatment with MFB.

### MFB significantly decreases the CD44^+^CD24^−/low^ population and mammosphere formation of breast cancer cells

**Figure 4:**
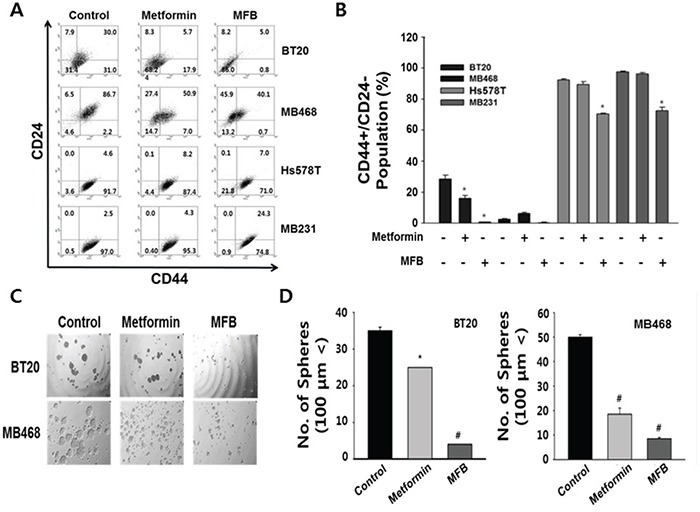
MFB significantly decreases the CD44^+^CD24^−/low^ population and mammosphere formation in breast cancer cells Subconfluent BT20, MDA-MB-468, Hs578T, and MDA-MB-231 cells were treated with 10 mM of metformin or MFB for 48 h, stained with FITC-conjugated CD44 antibodies and PE-conjugated CD24 antibodies, and subjected to flow cytometry. **A.** Representative flow cytometry dot plots are presented for the expressions of the CD44 and CD24 cell markers. Each numeric value indicates the cell SD **B. C** and **D.** Mammosphere-like structures formed by BT20 and MDA-MB-468 cells grown in sphere medium for 5 days in the absence or presence of 10 mM metformin or MFB were imaged using phase-contrast microscopy (100 x magnification, C) and graphs as means ± SD (D). The data shown are representative of three independent experiments. Symbols: *, *p* < 0.05; ^#^, *p* < 0.01 compared to controls.

Next, we explored whether MFB treatment significantly inhibited the population of breast cancer cells that were CD44^+^CD24^−/low^, which is a feature of breast CSCs [33]. Using flow cytometric analysis, we assessed the distribution of these cell-surface stem cell markers in TNBC cells following treatment (48 h) with 10 mM MFB or metformin. The percentage of BT20 cells exhibiting the putative stem-like CD44^+^CD24^−/low^ immunophenotype decreased from 31 ± 3% (in untreated control cells) to 0.8 ± 0.2% and 17.9 ± 4% following exposure to 10 mM MFB and metformin, respectively (Figure 4A, top panel). MFB treatment also significantly reduced the CD44^+^CD24^−/low^ cell populations in MDA-MB-468 cells (from 2.2% to 0.7%), Hs578T (from 91.7% to 71.0%), and MDA-MB-231 cells (97.0% to 74.8%) (Figure 4A and 4B).

To examine whether MFB could inhibit the properties characteristic of breast CSCs or circulating tumor cells (CTCs) more effectively than metformin, we evaluated the number of spheres generated in the absence or presence of metformin or MFB (10 mM) for 48 h. In BT20 and MDA-MB-468 cells, MFB treatment blocked sphere formation and significantly reduced the mean number and size of the formed spheres (Figure 4C and 4D). Remarkably, the effects of MFB on sphere formation were observed at a lower concentration (50 μM, which slightly reduced cell viability) than that required to obtain the same results from metformin treatment (data not shown), these results show that MFB inhibits the breast CSC population.

### MFB more effectively inhibits the CD44^+^ CD24^−low^ cells (putative CSCs) in heterogeneous breast cancer cell populations

**Figure 5:**
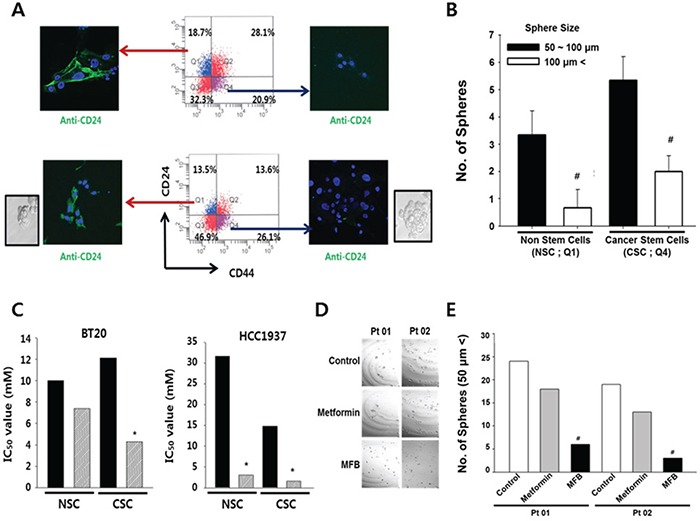
Compared to metformin, MFB more potently targets CD44^+^CD24^−/low^ breast cells (putative CSCs) in heterogeneous breast cancer cell populations **A.** Both breast CSCs (CD44^+^CD24^−/low^) and their counterpart non-stem cancer cells (NSCs) were isolated from subconfluent BT20 (top) and HCC1937 (bottom) cells by FACS, and immunofluorescence was used to assess the expression of CD24 (green fluorescence). **B.** Mammosphere formation of BT20 was analyzed, and the mammosphere formation efficacy (MSFE) was calculated in terms of sphere size and number after 3 days, using FACS. Q1 depicts the quadrant containing the CD44^−/low^CD24^+^ cells, while Q4 contained the CD44^+^CD24^−/low^ cells. **C.** Cell viability was measured in sorted cell populations exposed to metformin (10 mM) or MFB (0.01-100 mM, 48 h), and IC_50_ values were calculated for NSCs and CSCs. **D** and **E.** Mammosphere-like structures derived from cells obtained from two breast cancer patients (Pt 01, Pt 02) were analyzed in sphere medium for 5 days in the absence or presence of 10 mM metformin or MFB (100 x magnification) (D). The numbers of spheres (> 50 μm) are summarized in (E). Symbols: *, *p* < 0.05; ^#^, *p* < 0.01 compared to controls. The data shown are representative of three isolated experiments.

To examine whether the effect of MFB on breast cancer cells might be inhibited by the expression levels of stemness-related molecules, we used immunofluorescence staining and FACS to isolate CSCs (CD44^+^CD24^−/low^) and their counterpart non-stem cancer cells (NSCs; CD44^−/low^CD24^+^) from BT20 and HCC1937 TNBC cells. We confirmed the expression of CD24 (green fluorescence) in both cell lines (Figure 5A) and further validated that the size and number of formed spheres was higher in the CSC populations of both cell lines, compared with their NSC counterparts (Figure 5B). The sorted cells were then exposed to metformin or MFB (0.01-100 mM for 48 h), cell viability was measured, and IC_50_ values were calculated. In BT20 TNBC cells, the cytotoxic effect of MFB was dramatically higher in CD44^+^CD24^−/low^ breast CSCs (IC_50_, 4.2. mM) compared to CD44^−^CD24^+/low^ breast NSCs (IC_50_, 7.8 mM), whereas the IC_50_ value for metformin was somewhat higher in BT20-derived CSCs than in BT20-NSCs (Figure 5C, left). Conversely, in HCC1937 TNBC cells, the IC_50_ value for metformin was significantly lower in CSCs (14.8 mM) than NSCs (32.0 mM), whereas the values for MFB were similar in CSCs and NSCs (IC_50_ < 3.0 mM). This suggests that MFB inhibited breast CSCs more effectively than breast NSCs.

To begin examining the potential clinical relevance of MFB treatment, we treated cells derived from two breast cancer patients with metformin or MFB (10 mM), and examined sphere formation. We found that MFB treatment decreased sphere formation to a significantly higher degree than metformin (Figure 5D and 5E). Together, our results suggest that MFB could hold therapeutic promise for targeting breast CSC populations.

### MFB shows a more potent anti-tumor effect than metformin *in vivo*

**Figure 6:**
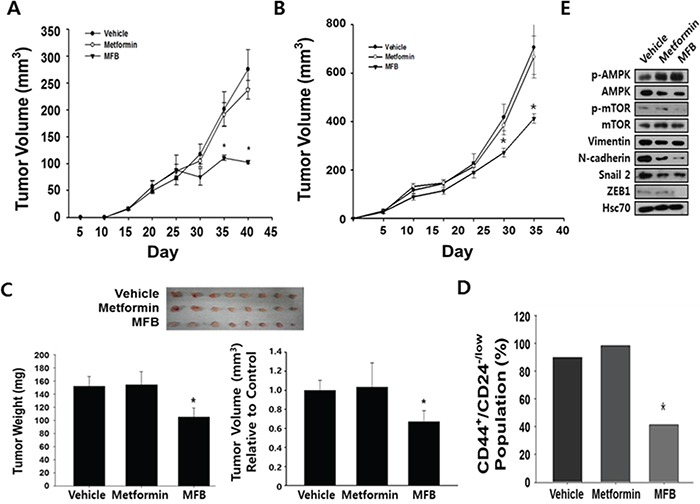
Compared to metformin, MFB shows more potent anti-tumor effects *in vivo* NDY-1 **A.** and MDA-MB-231 **B.** cells were injected into the mammary fat pads of NOG mice, and tumor-bearing mice (n = 6 per group) were treated daily with i.p. injections of metformin or MFB (250 mg/kg body weigh) for 3 weeks. Treatment with vehicle, metformin, or MFB was started when the average tumor volume was 50 mm^3^. Tumor volume was measured at the indicated time points. Data are representative of three independent cohorts of mice. **C.** MDA-MB-231 cells were pretreated with vehicle, metformin, or MFB (10 mM) for 16 h, and then injected into the orthotopic mammary fat pads of severely immunocompromised NOD/SCID IL2Rg (null) (NOG) mice (1 × 10^4^ cells/mouse). Gross morphological examination of representative xenograft tumor tissues excised from each group at 2 months post-injection (n = 8 per group, upper). MFB-pretreated cell-injected xenograft tumors had significantly lower weights and tumor volume than xenograft tumors generated using untreated and metformin-pretreated cells (bottom). **D.** The CD44^+/high^CD24^−/low^ CSC populations in NDY-1 derived xenograft tumors described in Figure 6B were collected, depleted of mouse cells, and analyzed using flow cytometry. **E.** The xenograft tumors described in (B) were collected and subjected to Western blot analyses with specific antibodies against AMPK, p-AMPK, mTOR, p-mTOR, EMT-associated proteins, and β-actin. Symbol: *, *p* < 0.05.

We investigated the ability of MFB to inhibit *in vivo* tumor growth, using xenograft mouse models in which MDA-MB-231 and NDY-1 cells were injected into the mammary fat pads of immunocompromised NOD/scid IL2Rg (null) (NOG) mice. Tumor-bearing mice were intraperitoneally (i.p.) injected with MFB or metformin (250 mg/kg) once a day for 21 days. We found significant decreases in the tumor growth, tumor volume (by ~ 40%), and tumor growth rate (which slowed time-dependently) in mice treated with MFB compared to those observed in mice treated with vehicle or metformin (which did not significantly differ in any parameter) (Figure 6A and 6B). These results demonstrate that MFB inhibits breast cancer cell growth more effectively than metformin *in vivo*, suggesting that it could be a promising therapeutic drug against TNBC.

Next, to compare the anti-tumor effects of metformin and MFB *in vivo*, MDA-MB-231 cells (1 × 10^4^) were pretreated *in vitro* with metformin or MFB for 16 h, and then injected orthotopically into immunocompromised mice. The mice were then evaluated for tumor initiation and growth. Although all mice showed initiation of solid tumor formation around the same time, those injected with MFB-pretreated cells formed tumors that were slightly smaller in tumor volume and weighed significantly less compared to tumors derived from control or metformin-treated cells (Figure 6C). Finally, we analyzed the CD44^+^ and CD24^−/low^ populations in mouse-cell-depleted cancer cells isolated from obtained from vehicle-, metformin- or MFB (250 mg/kg)-treated xenograft tumors. Significantly fewer CD44^+^CD24^−/low^ breast CSCs were found in MFB-treated NDY-1 xenograft tumors (Figure 6B) compared to vehicle- or metformin-treated xenograft tumors (Figure 6D). To identify the potential underlying mechanisms for this effect, we subjected xenograft tumor tissues to Western blot analyses against p-AMPK, p-mTOR and mesenchymal phenotype-associated proteins (i.e., slug, vimentin, N-cadherin, and ZEB1). The level of p-AMPK (and thus the activity of AMPK) was higher, while the levels of p-mTOR and the tested mesenchymal markers were lower in MFB-treated cell-derived xenograft tumors compared to vehicle- or metformin-treated cell-derived xenograft tumors (Figure 6E). Given that mesenchymal markers might directly drive the emergence of breast CSC phenotypes, we confirmed that the ability of MFB to significantly repress the expression of mesenchymal markers, such as slug, vimentin, N-cadherin, and ZEB1, the decreased mesenchymal markers levels in MFB-pretreated cell-derived xenograft tumors could be expected (Figure 6E).

Collectively, our results indicate that, compared to metformin, MFB yields improved anti-neoplastic activity by more specifically and effectively targeting breast CSCs and impairing their entry into (or progression through) S phase.

## DISCUSSION

This study reveals that metformin-butyrate (MFB), a derivative of metformin, could be a promising therapeutic agent against breast cancer. Our *in vitro* and *in vivo* experiments show that, compared to metformin, MFB appears to more effectively impair S phase entry and/or progression through G2/M phase and decrease mammosphere formation, especially in the CD44^+^CD24^−/low^ population that resembles breast CSCs. Emerging evidence from epidemiologic and preclinical studies suggests that metformin exerts anticancer activity [1–4, 34], but the clinical translation of this finding has been limited by the high concentrations of metformin required to obtain anticancer activity [13, 31, 34]. It is uncertain that whether this high concentration of metformin can be achieved without adverse effect in humans. Thus, structural analogs of metformin should be designed, synthesized and tested for their ability to deliver better anticancer activity and target specificity than metformin. Here, we report the development of a novel metformin analog with superior anti-neoplastic effects. We screened breast cancer cell lines for decreased cell viability in the presence of various analogs of metformin *in vitro*, and found that, compared to metformin, MFB exerted a stronger dose-dependent growth-inhibitory effect on various breast cancer cell lines. Metformin is known to inactivate mTOR via activation of AMPK, thereby directly inhibiting cell growth and tumorigenesis [9, 31]. Although the precise underlying mechanism(s) have not yet been fully elucidated, increasing evidence indicates that the P13K/Akt/mTOR pathway plays a crucial role in the survival and proliferation of cancer cells [9, 11, 15, 32, 35]. Therefore, it is clinically important that, compared to metformin, MFB markedly activated AMPK and inactivated mTOR with greater potency in all tested breast cancer cells *in vitro* and *in vivo*.

Metformin has been shown to block the cell cycle at G0/G1 phase without inducing apoptosis in prostate cancer cells [36], whereas it reportedly stimulates apoptosis in pancreatic cancer cells [37]. This apparent discrepancy may reflect variations in the utilized experimental conditions and/or it could indicate that metformin has cell-type-specific functions. Some studies have suggested that the responsiveness of a cell to metformin is related to its p53 status [38]. In the present study, however, the p53 status did not appear to affect the sensitivity of breast cancer cells to MFB-induced apoptosis. Whereas metformin caused G1 arrest and apoptosis (at higher doses) without altering the S phase population, MFB not only triggered G1 arrest by decreasing cyclin D expression, it also specifically inhibited S phase entry/progression, presumably via decreased expression of cyclin E, cdc2 and A. Moreover, it triggered G2/M arrest, perhaps by enhancing the expression of Wee2 (Wee1B), which is responsible for the inhibitory phosphorylation of CDK1 (Tyr15) and subsequent inactivation of the CDK1/cyclin B complex [39]. Therefore, there are critical differences in the cytotoxic effects of MFB and metformin. MFB was associated with enhanced G2/M arrest without any residual S phase population, whereas metformin enhanced S phase but did not appreciably enhance the G2/M phase population.

Due to the success of endocrine therapy, the mortality of breast cancer patients with estrogen receptor (ER)-positive tumors has significantly decreased in recent years. In contrast, triple-negative breast cancers (TNBCs, which lack clinical expression of estrogen and progesterone receptors while showing overexpression of the HER-2 receptor) cannot be treated with current endocrine or HER2-targeting therapies. They also tend to relapse early and metastasize, leading to poor patient survival [40–43]. Therefore, it is urgent that we develop new therapeutic reagents for TNBC patients. Some *in vitro* studies have demonstrated that the antitumor effect of metformin is most prominent for TNBC, but that this effect requires a very high dose of the drug [15, 44, 45]. Consistent with these previous reports, we observed that metformin was preferentially cytotoxic to TNBC cell lines (mean IC_50_ values of 31.2 and 17.2 mM in non-TNBC and TNBC, respectively). In contrast, MFB was not only more effective against breast cancer than metformin, it was equally effective against non-TNBC and TNBC cells (mean IC_50_, 4.3 and 4.1 mM, respectively). Furthermore, MFB was more effectively therapeutic in combination with docetaxel or cisplatin, and MFB, but not metformin, effectively targeted the CD44^+/high^CD24^−/low^ population sorted from tumor cells prepared from mouse xenografts. The no response of the cells to metformin might be possible adaptation or trans-differentiation of tumor cells to be less sensitive to metformin without losing the sensitivity to MFB.

TNBC is characterized by a larger subpopulation of stem-like cells compared to other molecular subtypes [46, 47]. The existence of these breast CSCs may explain the failure of high-dose chemotherapy in patients with distant metastases or locally advanced tumors. Therefore, effective therapy of TNBC could benefit from strategies aimed at eliminating CSCs. Interestingly, metformin was preferentially cytotoxic to CSCs or stem cell-like cells relative to non-CSCs. Hirsch et al. recently demonstrated that sphere forming, self-renewing breast CSCs appear to exhibit sensitivity to metformin [5], while Alejandro et al. reported that CSCs can promote mammosphere formation [26]. In the present work, we found that MFB (even at relatively low concentrations compared to the dosage of metformin) dramatically suppressed the sizes and numbers of mammospheres formed by cell lines and primary epithelial cells. Our *in vitro* and *in vivo* studies also showed that, compared to metformin, MFB more effectively targeted the population of cells with the CD44^+^CD24^−/low^ surface marker distribution, which is consistent with breast CSCs [33]. Therefore, this study reveals that the cytotoxicity of MFB against breast cancer cells has unique features compared to the response triggered by its parent compound, metformin-HCl. Future work is needed to better understand the molecular mechanism(s) through which MFB promotes the death of cancer (stem) cells. However, it is clinically interesting to note that MFB could block S phase entry/progression following G1 arrest, cause significant G2/M phase arrest, and target the CD44^+/high^CD24^−/low^ population that may be representative of CSCs.

In sum, we herein show that the metformin derivative, MFB, blocks breast cancer cell growth and cell cycle progression via G1 and G2/M arrests, whereas metformin causes only G1 arrest. In addition, MFB blocks growth and decreases survival in the CD44^+^CD24^−/low^ cell population more effectively than metformin *in vitro* and *in vivo*. These findings suggest that MFB could be a promising new agent for treating breast cancer.

## MATERIALS AND METHODS

### Chemicals

Metformin (1,1-dimethybiguanides) and MFB were obtained from HanAll BioPharma Co., Ltd.

### Cell culture and mammosphere formation assay

The MCF7, MDA-MB-231, MDA-MB-436, and MDA-MB-157 cell lines were obtained from the American Type Culture Collection (ATCC; Manassas, VA, USA). The SK-BR-3, ZR75-1, Hs578T, BT20, HCC70, HCC1937, DU4475, HCC38, and T47D cell lines were obtained from the Korean Cell Line Bank (KCLB, Seoul, Korea). BT474 cells were kindly provided by Dr. Incheol Shin (Hanyang University, Seoul, Korea). These cells were cultured as described in the ATCC website (www.atcc.org). The MDA-MB-231, MDA-MB-436, MDA-MB-453, MDA-MB-468, and Hs578T cell lines were cultured in DMEM (Gibco, CA, USA) containing 10% fetal bovine serum (FBS; Invitrogen, Carlsbad, CA, USA) and 1% penicillin/streptomycin (Gibco). SBCC-1 and SBCC-2 cells (from invasive breast cancer) and the NDY-1 cell line (breast sarcoma) were established in our laboratory, as previously described [48]. All of the other cell lines were grown in RPMI 1640 supplemented with 10% FBS and 1% penicillin/streptomycin. Cells were maintained at 37°C in a humidified atmosphere of 95% air and 5% CO_2_, and periodically screened for mycoplasmic contamination.

### Mammosphere culture and mammosphere-forming efficiency (MSFE)

A mammosphere formation assay was performed to assess the self-renewal capacity of CSCs. Single-cell suspensions of breast cancer cells were plated at 1,000 cells/mL in serum-free DMEM:F12 medium (3:1 ratio) supplemented with 20 ng/mL epidermal growth factor (EGF; Invitrogen), 20 ng/mL basic fibroblast growth factor (bFGF; Millipore, Temecula, CA, USA), 10 ng/mL leukemia inhibitory factor (LIF, Millipore), B27 supplement (Invitrogen) and antibiotic-antimycotic (Invitrogen). Under these conditions, cells grew as nonadherent spherical clusters. The medium was replenished every 3~4 days, and cells were passaged weekly. After 7 days of incubation with different concentrations of metformin or MFB, the formed spheres were collected by centrifugation at 300 x *g* for 5 min and counted with an inverted phase-contrast Axiovert 25 microscope (Zeiss, Germany). Mammosphere-forming efficiency (MSFE) was calculated as the number of sphere-like structures (>100 μm diameter) formed by day 7 divided by the original number of seeded cells and expressed as the mean percentage (± standard deviation, SD).

### Cell viability assay and combination treatment

The cytotoxic effects of metformin or MFB on the various cell lines were evaluated using a CellTiter-Glo Luminescent Cell Viability Assay Kit (Promega). Briefly, cells were plated in triplicate (1,000 cells per well; 96-well plates) and incubated in medium containing 10% FBS. After 24 h, the complete medium was replaced with test medium containing the vehicle control or various doses of metformin or MFB for 48 h at 37°C. The Cell-Titer-Glo assay buffer was then added, and cell viability measured according to the provided protocol. To assess cytotoxicity, CD44^+^CD24^−/low^ and CD44^−/low^CD24^+^ cells were seeded as described above, treated for 48 h with 10 mM metformin or MFB, and examined as described above.

### Flow cytometric analysis and fluorescence-activated cell sorting (FACS)

Cells were dissociated from spheroids or monolayers with trypsin-EDTA solution. Suspended cells were collected by centrifugation and washed with a flow cytometry buffer comprising PBS containing 0.1% bovine serum albumin (BSA; Bovogen Biological, Melbourne, Australia) and 0.05% sodium azide. Cells (5 × 10^5^) were stained using the recommended concentrations of fluorochrome-conjugated monoclonal antibodies against human CD24 and CD44 (PharMingen Biosciences, San Jose, CA) for 20 min at RT in the dark. After staining, cells were washed with 3 ml of flow cytometry buffer and resuspended in the same buffer. Flow cytometric analysis was performed by analyzing 5,000 events on a FACSCalibur flow cytometer (BD Biosciences). For FACS, single cells were suspended in 1% FBS/PBS buffer labeled with anti-CD44 (FITC-labeled) and anti-CD24 (PE-labeled) and isolated using a FACSAria flow cytometer (BD Biosciences). Routinely, more than 88% of the sorted cell population was CD44^+^CD24^−/low^.

### Apoptosis analysis

Apoptosis was detected using an Annexin V-FITC Apoptosis Detection Kit (BD PharMingen) according to the manufacturer's instructions. In brief, drug-treated cells were washed with PBS, mixed with binding buffer, and then incubated with Annexin V-FITC for 20 min at RT. The cells were incubated with propidium iodide (PI) for 10 min on ice in the dark, and apoptotic cells were measured by FACS. To further confirm apoptosis, we measured caspase-3/7 activity with a Caspase-Glo 3/7 assay (Promega, Madison, WI). Briefly, the cells were seeded in 96-well plates under the indicated treatment conditions, and reagents from the assay kit were added to the culture medium for 24 h. At the end of the incubation period, caspase-3/7 activity was measured with a luminometer.

### Western blotting and antibodies

Cells were washed twice with ice-cold PBS, and total cell lysates were prepared in lysis buffer (20 mM Tris-HCl, pH 7.5, 150 mM NaCl, 1 mM EDTA, 1 mM EGTA, 1% Triton X-100, 2.5 mM sodium pyrophosphate, 1 mM β-glycerophosphate, 1 mM Na_3_VO_4_, 1 mM dithiothreitol, 10 μg/ml leupeptin, 10 μg/ml aprotinin, 1 mM PMSF). Protein concentrations were measured with the Bradford assay using a Bio-Rad Protein Assay kit (Bio-Rad Laboratories, Hercules, CA), according to the manufacturer's instructions. Equal amounts of lysed proteins were separated by 6 ~ 10% SDS-PAGE, and the protein bands were electrotransferred to Hybond-ECL nitrocellulose membranes (Amersham Bioscience, Buckinghamshire, UK). The blots were blocked with blocking buffer (5% skim milk in TBS-T) for 1 h and then incubated overnight at 4°C with mouse monoclonal antibodies against p-AMPK, p-mTOR, snail 2, vimentin, N-cadherin, ZEB1, or β-actin (Sigma). The blots were then washed three times in TBS-T, and incubated with peroxidase-conjugated Affinipure rabbit anti-mouse IgG (1:5000 dilution; Jackson ImmunoResearch) or peroxidase-conjugated Affinipure mouse anti-rabbit IgG for 1 h at RT. The immunocomplexes were washed with TBS-T (three times, 5 min each), and then visualized by enhanced chemiluminescence (Amersham Biosciences).

### Sample preparation for the antibody array

MDA-MB-231 cells were treated with 10 mM metformin or MFB for 24 h, The protein was extracted using protein extraction buffer (Fullmoon Biosystems, Sunnyvale, CA) containing 1% protease inhibitor cocktail (Sigma), 1% phosphatase inhibitor cocktail (Sigma) and lysis beads (Fullmoon Biosystems). The extracted protein solution was purified using the gel matrix column provided in the antibody array assay kit (Fullmoon Biosystems). The column was vortexed for 5 seconds, hydrated for 60 minutes at RT, and centrifuged at 750 x g for 2 minutes. After centrifugation, the column was placed into a collection tube, and 100 ul of purified protein was collected by centrifugation at 750 x g for 2 minutes. The concentration of the purified sample was measured with a BCA protein assay kit (Pierce, Rockford, IL) and a NanoPhotometer (Implen, UK).

### Antibody array assay

The obtained protein sample (50 ug) was brought to 75 ul with labeling buffer, treated with 3 ul of 10 ug/ul biotin/DMF solution, and incubated at RT for 1 h with mixing. Stop reagent (35 ul) was added, and the sample was incubated at RT for 30 min with mixing. The antibody microarray slide (Fullmoon Biosystems) was shaken (55 rpm) with 30 ml of blocking solution in a petri dish for 1 h at RT. After blocking, the slide was rinsed with Milli-Q grade water. The labeled sample was mixed with 6 ml of coupling solution, and the blocked array slide was shaken with the coupling mixture (60 rpm) for 2 h at RT in a coupling dish. The slide was then washed six times with 30 ml of washing solution in a petri dish with shaking (55 rpm) for 5 minutes, and then rinsed with Milli-Q-grade water. For detection, 30 ul of 0.5 mg/ml Cy3-streptavidin (GE Healthcare, Chalfont St. Giles, UK) was mixed with 30 ml of detection buffer and shaken (55 rpm) with the coupled array slide for 20 minutes at RT. The slide was washed six times with 30 ml of washing solution in a petri dish (55 rpm, 5 minutes each), and rinsed with Milli-Q-grade water.

### Antibody array data acquisition and analysis

Slides were thoroughly dried, and scanned using a GenePix 4000B scanner (Axon Instrument, USA). Each obtained scan image was gridded and quantified using the GenePix Software (Axon Instrument). The numeric data were analyzed using the Genowiz 4.0 software (Ocimum Biosolutions, India), and protein data were annotated using the UniProt DB (www.uniprot.org).

### *In vivo* xenograft and tumor initiation experiments

Seven- to eight-week-old female NOD/scid IL2Rg (null) (NOG) mice were purchased from Jackson Laboratory (Bar Harbor, ME) and maintained in accordance with the standards of the Seoul National University Hospital Animal Ethics committee (Seoul, Korea). Cells were injected into the inguinal mammary fat pads of mice, and the engrafted mice were inspected twice a week for tumor appearance, using visual observation and palpation. At a tumor diameter of 1 cm or 2-3 months post-transplantation, mice were sacrificed and analyzed. For the tumor initiation study, MDA-MB-231 cells were treated with 10 mM of metformin or MFB for 16 h prior to transplantation. Tumor sizes were measured using digital calipers and tumor volumes (width^2^x length)/2 were calculated.

### Statistical analysis

Graphs were generated and quantitative results were compared with the paired Student's t test using the Sigma Plot software (Statistical Solutions Ltd., Cork, Ireland). *P* values less than 0.05 were recognized as statistically significant.
