# Correction: Enhanced anti-tumor activity and cytotoxic effect on cancer stem cell population of metformin-butyrate compared with metformin HCl in breast cancer

**DOI:** 10.18632/oncotarget.27986

**Published:** 2021-06-08

**Authors:** Kyung-Min Lee, Minju Lee, Jiwoo Lee, Sung Wuk Kim, Hyeong-Gon Moon, Dong-Young Noh, Wonshik Han

**Affiliations:** ^1^ Biomedical Research Institute, Seoul National University Hospital, Seoul 110-744, Republic of Korea; ^2^ Cancer Research Institute, Seoul National University College of Medicine, Seoul 110-744, Republic of Korea; ^3^ Department of Surgery, Seoul National University College of Medicine, Seoul 110-744, Republic of Korea; ^4^ Hanall Biopharma Co., Ltd., Seoul 138-922, Republic of Korea

**This article has been corrected:** Due to errors during figure assembly, the data on β-actin in BT20, MB231, and Hs578T cells in Figure 2A, and the data on Hsc70 in BT20, MB231, and Hs578T cells in Figure 3C, are mistakenly identical. However, the original data is unavailable. In light of this, a new set of data was submitted for this correction as shown in the updated Figure 3 below. At the request of *Oncotarget*, the Seoul National University Research Integrity Committee has investigated the case and concluded: This error is not serious, as the above data had no direct impact on the results and conclusions of the study....The action is considered an inappropriate research practice per Article 12-1 of the school’s Research Ethics Guidelines ‘Falsification of research data due to error without serious impact on the study,’ and the degree of violation of research integrity is determined to be relatively minor in light of the details of this case as described above.

Original article: Oncotarget. 2016; 7:38500–38512. 38500-38512 https://doi.org/10.18632/oncotarget.9522

**Figure 3:**
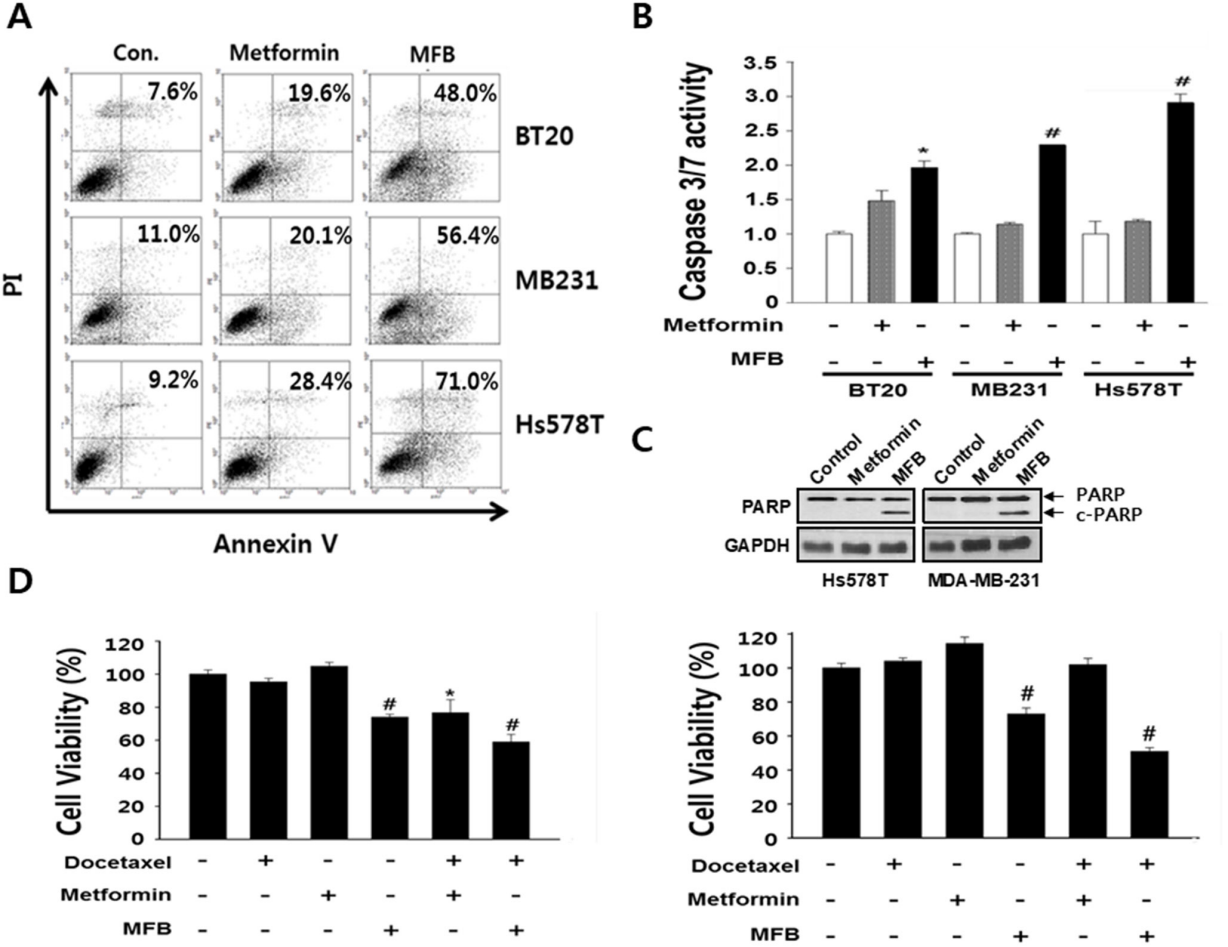
MFB induces the apoptosis in breast cancer cells. (**A**) Subconfluent BT20, MDA-MB-231 and Hs578T cells were treated with 10 mM metformin or MFB for 48 h and analyzed for cellular apoptosis by Annexin V/PI staining followed by flow cytometry. Apoptosis were confirmed Caspase-3/7 activities using the Caspase-Glo 3/7 assay. (**B**) and c-PARP expression (**C**, **D**) BT20 (left) and MDAMB-231 (right) cells were treated with 10 mM metformin or MFB in the presence or absence of docetaxel (50 nM) or cisplatin (50 μM) or 48 h, and cell viability was analyzed using a CellTiter-Glo Luminescent Cell Viability Assay Kit. Data are expressed as the means ± SD of triplicate experiments. Symbols: ^*^, *p* < 0.05; ^#^, *p* < 0.01 compared to controls. The data shown are representative of three different experiments.

